# Identifying what works for whom: Implementation outcomes following *iLookOut*, a child abuse identification and referral training program

**DOI:** 10.1017/cts.2023.628

**Published:** 2023-09-15

**Authors:** Whitney C. Barnett, Carlomagno C. Panlilio, Casey Mullins, Benjamin H. Levi, Kathryn L. Humphreys

**Affiliations:** 1 Department of Psychology and Human Development, Vanderbilt University, Nashville, TN, USA; 2 Department of Educational Psychology, Counseling, and Special Education, The Pennsylvania State University, State College, PA, USA; 3 Department of Psychology, University of Miami, Coral Gables, FL, USA; 4 Department of Humanities and Pediatrics, Penn State College of Medicine, Hershey, PA, USA

**Keywords:** Child abuse, child maltreatment, neglect, mandated reporter training, implementation outcomes, training evaluation

## Abstract

**Introduction:**

*iLookOut*, a web-based child abuse training for early childcare professionals (ECPs), has been shown to improve knowledge and attitudes related to correctly identifying and reporting suspected cases of child abuse. The overarching goal of the present study is to examine “what works for whom” for *iLookOut* in order to identify strategies for optimizing learner outcomes.

**Methods:**

This prospective study enrolled 12,705 ECPs who completed iLookOut (November 2014–December 2018). We used structural equation models to test whether learner demographic and professional characteristics were differentially associated with implementation outcomes (i.e., acceptability and appropriateness) and whether these mediated subsequent indicators of training effectiveness (i.e., gains in knowledge).

**Results:**

Consistent with previous research, individuals with lower baseline knowledge scores showed greater knowledge gains (*β* = −.57; *p* < .001). Greater knowledge gains were seen for learners who reported higher acceptability (*β* = .08; *p* < .001) or appropriateness (*β* = .14; *p* < .001). Implementation outcomes strongly associated with knowledge gains included acceptability for female learners and appropriateness for learners who had not completed high school or had >15 years of experience in childcare settings. Where mediation was found, for the majority of groups, appropriateness emerged as the driving mediator.

**Conclusion:**

Implementation outcomes emerged as important drivers of knowledge change for most groups. The *iLookOut* Core Training’s use of a multimedia learning environment, video-based storylines, and game-based techniques were endorsed by learners and correlated with increases in knowledge. Future work should explore why aspects of the *iLookOut* training are rated as less acceptable or appropriate by some groups and what changes would improve efficacy for low performing learners.

## Background

Child maltreatment, encompassing physical, sexual, and emotional abuse, and neglect, is an urgent public health problem in the USA. Annually, almost 8 million children are reported to child protective services (CPS) for potential child maltreatment, and nearly three quarters of a million children are determined to be victims of abuse or neglect [1]. Child maltreatment can have a devastating impact on individual health, significantly increasing the risk of cognitive impairment [2,3] and mental health disorders [3], including behavioral problems [2,4]. The adverse consequences of child maltreatment extend into adulthood by increasing individual risk for mental health issues and chronic health problems over one’s life course [5,6].

Over 12 million children in the USA, or approximately 60% of children under 5 years, are cared for by a nonparent on a regular basis (e.g., in a childcare center, or home-based care by a nonrelative) [7]. Young children are at highest risk for maltreatment and abuse-related fatalities [1]. Early childcare professionals (ECPs) spend substantial time with young children, often more than any other adult outside of their family. Therefore, this group is well positioned to identify signs of potential child maltreatment in young children. Further, ECPs are specifically identified as mandated reporters in all but three states (Indiana, New Jersey, and Wyoming), and in those three states all competent adults are designated as mandated reporters [8]. ECPs have a legal obligation to report suspected maltreatment. Yet, ECPs constitute less than one percent of referrals to CPS [1], suggesting that ECPs underreport child maltreatment [1,9,10]. Underreporting represents a potentially critical gap to ensure child safety, given that proper CPS intervention following a report remains a key deterrent for child maltreatment recurrence and failure to report often results in continued or worsening abuse [11,12].

*iLookOut for Child Abuse* (*iLookOut*) was designed to provide online education to improve ECP’s knowledge and awareness of signs and symptoms of child maltreatment, reporting requirements, and practical approaches to understanding how to use reasonable suspicion as a threshold for reporting child maltreatment. Multiple studies have shown that *iLookOut* is effective at changing knowledge and attitudes related to child maltreatment, both in randomized controlled trials and real-world settings [13–16].

However, these same studies indicate that not all participants demonstrate the same knowledge gains. Differential knowledge gain may be due to variation in how specific learners experienced the training. For example, how acceptable or appropriate they found the training. Implementation outcomes (e.g., acceptability, appropriateness, and feasibility) are indicators of a program’s success. Studies have documented these as essential features of a program’s ability to affect training outcomes (e.g., to improve knowledge) [17]. Importantly, implementation outcomes indicate how well a program has been deployed and received by its targeted end user, reflecting end users’ experience of the program’s relevance, suitability, delivery methods, and appropriateness of content [18].

Implementation science is still a relatively nascent field, concerned with understanding the translation of evidence-based programs and interventions into routine practice and identifying and addressing barriers that slow the uptake or impact the efficacy of evidence-based programs in real-world settings [18]. Proctor *et al*. [19] established a conceptual framework to define and operationalize an approach to evaluate a program’s implementation success. Specifically, they outlined a list of “implementation outcomes” distinct from clinical or training outcomes (e.g., knowledge gain), including measurement of a program’s acceptability, adoption, appropriateness, cost, feasibility, fidelity, penetration, and sustainability. This framework provides a useful approach to assess translation gaps of evidence-based programs and, ultimately, to provide guidance about how a program may be improved to better reach its goals. Additionally, implementation outcomes have been shown to differ based on setting as well as user characteristics [20,21].

*iLookOut’*s Core Training was developed to more effectively engage ECPs, compared to alternative mandated reporter trainings, through the use of a multimedia learning environment, video-based storylines, and game-based techniques [22,23]. It is important to understand how these training approaches have been received by the end user to identify whether implementation outcomes for *iLookOut* help to explain the program’s efficacy for increasing knowledge. Important information about the efficacy of these approaches can be used to improve similar programs and aspects of *iLookOut* for specific users.

Previous work investigating the efficacy of *iLookOut* found that training outcomes differ by learner characteristics. Specifically, that learner age, race, education, and employment status impact pre- to post-training changes in knowledge and attitudes [16]. Here, we aim to better understand whether specific program components and implementation outcomes differ for users based on professional (e.g., years of experience as a childcare worker, previous mandated reporter training) or demographic characteristics (e.g., age, gender, and educational attainment). Specifically, we will examine how the learner experience differs by user, whether these different experiences relate to efficacy of *iLookOut*, and ultimately, how *iLookOut*’s approach or content can be improved to increase learning for specific user groups. The present analyses include examining if prior training is related to acceptability of program components, how user profiles impact acceptability and knowledge gain, and whether targeted content based on pretest knowledge could yield shorter and more relevant training. Improved understanding of how user characteristics impact implementation and training outcomes could potentially enable individualized and responsive content, given that web-based trainings can be tailored (using branching logic) more easily than in-person training [24,25]. The present analysis also will examine the impact of *iLookOut’*s interactive approach to user engagement on perceived acceptability and appropriateness. Though implementation outcomes are commonplace for ensuring that evidence-based interventions are effective [26,27], few programs rigorously test implementation processes with the goal of understanding which program components are most effective for which users [18,28]. In particular, mandated reporter education programs are seldom evaluated and often lack an evidence-based approach [29].

The current study sought to evaluate the implementation outcomes for more than 12,000 ECPs in Pennsylvania who completed the *iLookOut* Core Training. We tested whether learner demographic and professional characteristics were differentially associated with acceptability and appropriateness, and whether these explain (mediate) subsequent indicators of training effectiveness (e.g., gains in knowledge). Our overarching goal is to examine “what works for whom” with *iLookOut* in order to identify strategies for 1) optimizing outcomes for learners of *iLookOut* and 2) providing generalizable insights (for related web-based interventions) regarding what does (and does not) influence outcomes for various learners.

## Methods

### Participants

Participants were ECPs in Pennsylvania who completed the *iLookOut* Core Training as part of an open-enrollment trial. Participants were not actively recruited to the study; however, *iLookOut* is one of more than a dozen approved mandated reporter trainings which are required for all ECPs in Pennsylvania. *iLookOut* is listed on the Pennsylvania Department of Human Services website and is available free of charge. Those who completed the training from November 2014 to December 2018 were prospectively enrolled into the current study (*n* = 12,705). Of the 12,705 who were enrolled, all participants contributed demographic and professional characteristic data (Table 1); 12,409 (98%) completed the knowledge measure both pre- and post-training; 12,693 (99%) contributed evaluation data following the training (item-level missingness for evaluation data is indicated in Table 2). No remuneration was provided for completion; however, ECPs were able to earn three hours of professional credit for completing the program. ECPs provided informed consent online prior to participating.

**Table 1.** Learner characteristics and knowledge change (*n* = 12,705)

Learner characteristics		*n* (*%*)
Gender		
	Female	11,309 (89%)
	Male	1,396 (11%)
Race/Ethnicity		
	White	8,737 (69%)
	Asian	274 (2%)
	Hispanic/Latino	768 (6%)
	Black/African American	2,614 (21%)
	Other	312 (3%)
Age range		
	18–29 years	6,181 (49%)
	30–44 years	3,329 (26%)
	≥45 years	3,195 (25%)
Education (highest level completed)		
	8^th^ grade	102 (1%)
	High school/GED	5,371 (42%)
	CDA	823 (7%)
	Associate degree	1,674 (13%)
	Bachelor’s degree	3,368 (27%)
	Graduate degree	1,367 (11%)
Years working as an early childhood practitioner		
	<5	8,158 (64%)
	6–10	1,837 (15%)
	11–15	957 (8%)
	>15	1,753 (14%)
Childcare work setting		
	Center-based: non-commercial	4,549 (36%)
	Center-based: commercial chain	3,170 (25%)
	Religious facility	1,239 (10%)
	Head Start facility	986 (8%)
	Home-based childcare	687 (5%)
	Other	2,074 (16%)
Prior mandated reporter training		
	Yes	8,430 (66%)
	No	4,275 (34%)
**Knowledge**		Median (IQR)
Knowledge score (baseline)		14 (12, 16)
Knowledge score (post-test)		17 (15, 19)
Knowledge change		3 (1, 5)

Note: CDA = Child Development Associate credential; GED = General Educational Development test.

Other race/ethnicities are: American Indian/Alaska Native, Native Hawaiian/Pacific Islander, and self-reported “other.”

Post-test knowledge score: *n* = 12,424; pretest knowledge score: *n* = 12,690; no missing demographic data.

**Table 2.** Item-level indicators of acceptability and appropriateness of *iLookOut*

	Yes	Disagree	Neutral	Agree
**Acceptability**	*n* (%)	*n* (%)	*n* (%)	*n* (%)
1. Ease of use (*n* = 12,652)	N/A	415 (3%)	1,177 (9%)	11,060 (87%)
2. Kept interest and attention (*n* = 12,625)	N/A	339 (3%)	1,063 (8%)	11,223 (89%)
3. Information was useful for my role as a mandated reporter of suspected child abuse/neglect (*n* = 12,616)	N/A	120 (1%)	502 (5%)	10,599 (95%)
4. Information was provided in a way that helped me learn (*n* = 12.604)	N/A	146 (1%)	591 (5%)	11,867 (94%)
5. Interactive storyline was a helpful way to learn about my responsibilities as a mandated reporter for suspected abuse/neglect (*n* = 12,619)	N/A	148 (1%)	555 (4%)	11,916 (94%)
6. The most helpful part of the…online training program was interactive scenarios	6,426 (51%)	N/A	N/A	N/A
7. The most helpful part of the… online training program was videos	7,022 (55%)	N/A	N/A	N/A
**Appropriateness** (learners could choose up to 3)				
“The most helpful part of the online training was…”				
8. Resource documents	4539 (36%)	N/A	N/A	N/A
9. Explanation of legal requirements	4422 (35%)	N/A	N/A	N/A
10. Background stories	2936 (23%)	N/A	N/A	N/A
11. Being available online at any time	3174 (25%)	N/A	N/A	N/A
12. Information about how to report child abuse/neglect	4290 (34%)	N/A	N/A	N/A

Notes: Program evaluation questions were given following the knowledge test after the training. Responses for each item were included for *n* = 12,705 unless otherwise noted.

### iLookOut training

The *iLookOut* Core Training is a free, online, evidence-based mandated reporter training. It was created in 2013–2014 by a multidisciplinary team of experts (in child abuse, instructional design, pediatrics, early childhood education, ethics, and online learning) under the aegis of the Center for the Protection of Children at the Penn State Children’s Hospital. *iLookOut* uses an interactive, video-based storyline involving five children to engage learners with the goal of improving knowledge related to child maltreatment and changing ECP behaviors for recognizing, responding to, and reporting suspected child maltreatment. As events unfold, the learner receives didactic information and engages in various interactive exercises related to the different forms of child maltreatment. Training content includes red flags that should raise concern, risk factors, protective factors, strategies for supporting children and their families, as well as the process and legal requirements for reporting suspected child abuse. The goal is to engage learners in real-life stories which empower them to practice and implement what they have learned to help protect and support children who may be experiencing child maltreatment [22].

### Measures

*Knowledge.* Knowledge was measured using a 23-item, true/false, expert-validated instrument previously described [13]. It measures individuals’ knowledge about what constitutes child maltreatment, risk factors for maltreatment, and legal requirements for reporting suspected maltreatment. Correct responses are scored as 1 and incorrect as 0 to provide a sum score for the 23-item knowledge measure, with a range of 0–23; higher scores indicate more knowledge about child maltreatment. Pre- and post-training question items were identical, but to reduce recall bias, their sequencing orders were changed between the pre- and post-tests. Knowledge change was the primary outcome used in models, calculated by subtracting pretraining score from post-training score. Change in knowledge score was included as a continuous variable in all models.

*Demographics*. Demographic and professional characteristics were collected pretraining using learner self-report, including gender, age, education, race/ethnicity, years of experience working as a childcare professional, learner’s work setting (e.g., religious facility, commercial center), and prior mandated reporter training. Each of these variables was dichotomized (dummy coded) prior to inclusion in analytic models.

*Implementation outcomes.* A post-training evaluation elicited feedback from learners about implementation outcomes. Constructs of acceptability and appropriateness were chosen based on Proctor’s conceptual framework 19, which aimed to define and operationalize a taxonomy for evaluating program implementation and success. Proctor defines *Appropriateness* as “the perceived fit, relevance, or compatibility” of a program for the end user and acceptability as the perception that it is “agreeable, palatable, or satisfactory” [19]. Learners reported on acceptability of the program, responding on a Likert scale (0 = *strongly disagree* to 6 = *strongly agree*) regarding whether the program was easy to use, if it kept their interest and attention, if information provided was useful to their role as a mandated reporter, whether information was provided in a way to help them learn, if the interactive storyline was helpful to learn about their responsibilities as a mandated reporter; and they indicated a yes or no to whether interactive scenarios or videos in the program were helpful. Appropriateness was reported as yes (1) or no (0) for whether each of the following were helpful: resource documents, explanation of legal requirements, background stories, the 24/7 online availability, or information on how to report child maltreatment. Likert response items were collapsed into three categories for inclusion in the final model: agree, neutral, and disagree based on responses.

### Analytic approach

**Figure 1.**
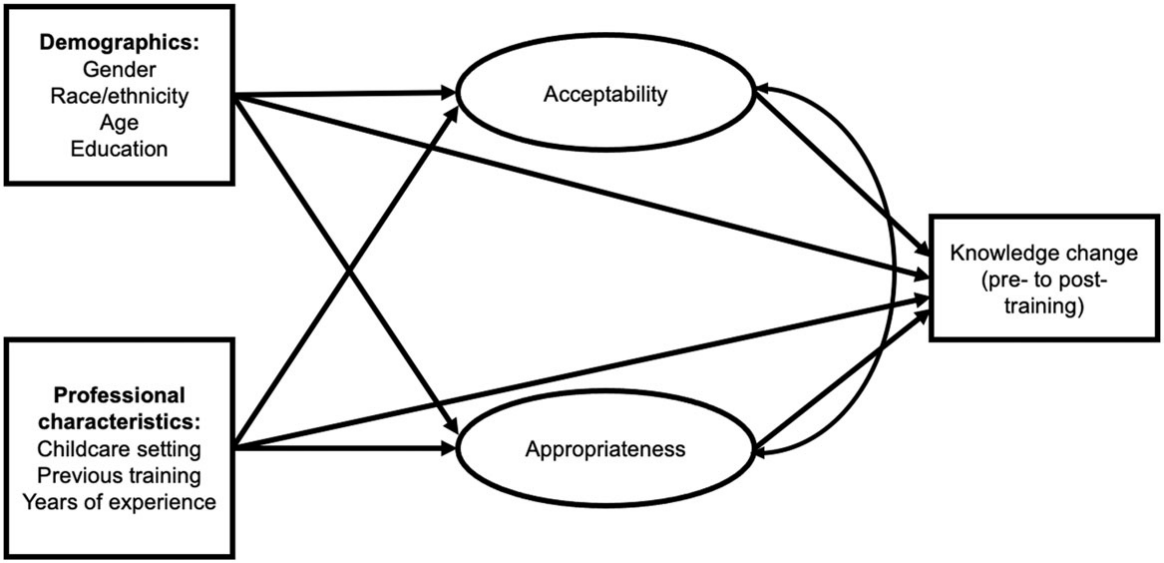
Conceptual diagram of the full structural equation model estimating direct and indirect effects.

Guided by Proctor’s model on implementation outcomes 19, the current study employed a multiple indicator multiple cause (MIMIC) structural equation modeling (SEM) to examine the mediating effects of implementation outcomes on ECP learner demographic/professional characteristics and knowledge change. A measurement model was specified using confirmatory factor analysis (CFA) to estimate the factor structure of acceptability and appropriateness, based on observed variables (described in Table 2) and to verify the underlying latent construct. Once confirmed by the CFA, latent factors for both acceptability and appropriateness were included in a MIMIC structural model, which estimates the effect exogenous variables have on latent variables that are not directly observable [30]. Acceptability and appropriateness were included concurrently in the MIMIC SEM to allow estimation of interrelationships between both latent variables (implementation outcomes) and observed variables (demographic/professional characteristics and knowledge change; Fig. 1). Once an appropriate measurement model was selected, the full latent variable path model (i.e., MIMIC SEM) was specified and included all hypothesized direct and indirect relationships, adjusting for baseline knowledge scores (see Fig. 1 for a conceptual diagram of the full MIMIC SEM model). MIMIC model was used in the main analysis of the study in order to examine the effects of demographic and professional characteristics as covariates in the CFA and SEM models [31]. The MIMIC CFA and SEM analyses were conducted using *Mplus* v.8.5 using diagonally weighted least squares estimation with theta parameterization to account for inclusion of categorical variables in the model [31].

Model fit was evaluated using root-mean-square error of approximation with its 95% confidence interval (RMSEA ≤0.06), along with comparative fit index (CFI ≥0.90) and model χ^2^ [32]. In lieu of a Sobel test, which is inappropriate with violations of normality assumptions, we used bias-corrected bootstrapped confidence intervals to examine the significance of the indirect effects within the model [33,34]. Additionally, prior to running mediation models, we evaluated missingness in our dataset to test whether data employed in the covariance matrix were missing completely at random (MCAR) using Little’s MCAR test [35].

*Mediation.* A mediation effect, or indirect effect, represents when a third variable operates as a pathway through which an exogenous variable affects an endogenous variable [36]. For the present study, we estimated indirect effects separately for both acceptability and appropriateness for each category of learner (demographic and professional characteristics), as well as the total indirect effect through both constructs (acceptability plus appropriateness), and lastly total effect (direct and indirect effects) on knowledge change (Fig. 1). The aim was to investigate whether implementation outcomes differentially mediate relationships for specific learner groups and pre- to post-training knowledge change to better understand how the training program works (and/or may be modified) to improve knowledge outcomes.

## Results

### Baseline analyses

Demographic and professional characteristics of learners completing the training are presented in Table 1. A majority of the participants identified as female (89%), White (69%), had completed prior mandated reporter training (66%), and had worked less than 5 years as an early childcare professional (64%). Median baseline knowledge score was 14/23 (IQR 12, 16), and median post-training knowledge score was 17/23 (IQR 15, 19; Table 1). Learner responses to item-level indicators of appropriateness and acceptability are reported in Table 2. A large majority of learners reported that the program was easy to use (87%), that it kept their interest and attention (89%), that information was useful for their role as a mandated reporter (95%), that information was provided in a way that helped them to learn (94%), and that the interactive storyline was helpful for learning (94%). When asked to identify up to three components of the training that were most helpful, a slight majority identified *iLookOut*’s interactive scenarios (51%) or videos (55%), and a minority identified resource documents (36%), explanation of legal requirements (35%), background stories (23%), being available online at any time (25%), or how to report child maltreatment (34%) (Table 2).

Lower baseline knowledge scores were seen for learners who identified as Asian, Hispanic, Black, and other racial or ethnic minorities (compared to White learners), those whose highest level of education was high school or lower, as well as the youngest learners (18-29 years old), had<6 years’ experience as an ECP, had no prior mandated reporter training, or who worked in a religious early childhood program setting (Appendix Table 1). In general, consistent with previous research [15], individuals with a lower baseline knowledge score showed greater knowledge gains (*β* = −.57, 95%CI: −.59, −.56, *p* < .001; Appendix Table 2). Greater knowledge gains were seen with learners who rated the training more highly for acceptability (*β* = .08, 95%CI: .06, .11, *p* < .001) or appropriateness (*β* = .14, 95%CI: .11, .16, *p* < .001; Appendix Table 2).

**Figure 2.**
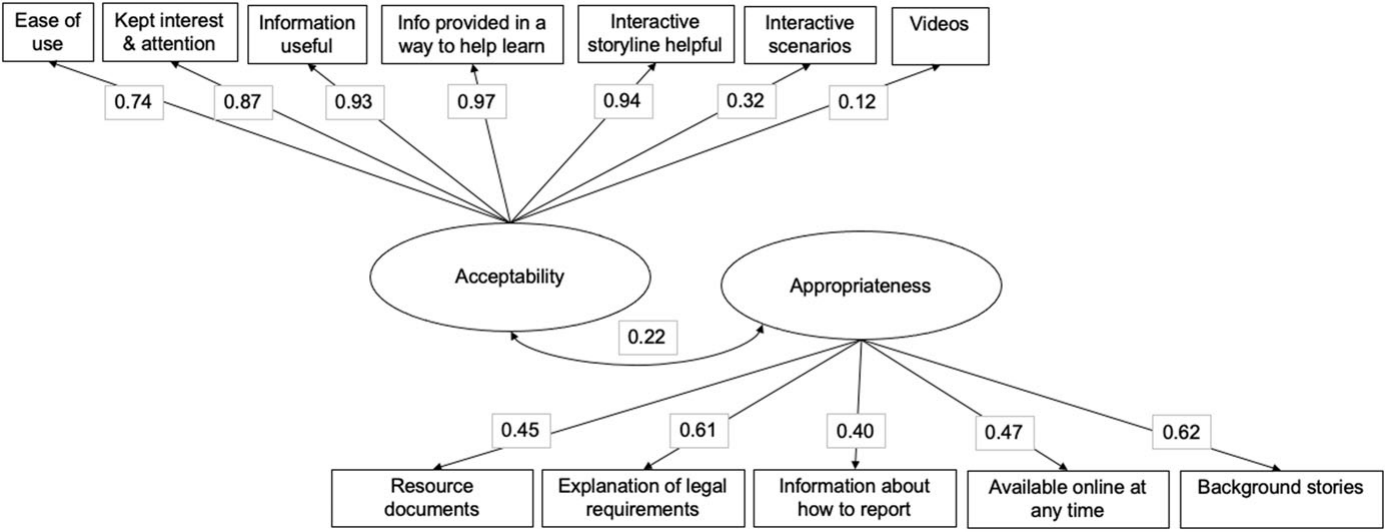
Confirmatory factor analysis of acceptability and appropriateness of *iLookOut. Note:* Figure depicts factor loadings (correlation) between the item-level indicator of either acceptability or appropriateness. All loadings are standardized; all paths are significant.

Prior to running the full mediation models, we examined the factor structure of acceptability and appropriateness using CFA models. A single factor model was used for both implementation outcomes. See Fig. 2 for the visual depiction of item-level indicators of acceptability and appropriateness with standardized loadings. All were statistically significant, and model fit was high (CFI = .98; RMSEA = .05).

Results of Little’s test indicated that missing data mechanism did not meet MCAR assumptions: χ2 (2, 12,705) = 235.09, *p* < 0.001; Little, 1988). Missingness for one item-level indicator of acceptability (interactive storylines were most helpful) was associated with learner’s age and was therefore included in the final measurement model to control for possible effects of age on this item. The resulting estimates were nonsignificant, providing minimal evidence for missing at random (MAR) to proceed with the main analyses.

### Results of path model by learner characteristic

Results of the MIMIC SEM showed good data-model fit (CFI = .97; RMSEA = .03). See Figure 1 for a visual depiction of the SEM model and Table 3 for standardized path coefficients. All direct, indirect, and total effects for each path estimated are presented in Appendix Tables 2–7. Table 3 provides a summary of estimated direct effects, indirect effects via appropriateness and acceptability, and total indirect effects for learner characteristics and knowledge change (summarizing Appendix Tables 2, 5, and 6). Table 4 also summarizes these results and provides a narrative summary of mediation findings by learner characteristic. Paths to test for mediation were estimated concurrently in a single model and adjusted for learner demographic and professional characteristics, as well as baseline knowledge scores. SEM results are reported separately below for each demographic or professional characteristic investigated.

**Table 3.** Standardized path coefficients: direct and indirect effects

		Direct effects	Indirect effects		
		Knowledge	via Acceptability	via Appropriateness	Total indirect
Gender: Female		−.01	.01***	.004	.01***
Race/ethnicity (Reference: White)					
	Asian	−.06***	.002	.004	.01*
	Hispanic	−.08***	.01**	.01**	.01***
	Black	−.16***	.002	.01**	.01**
	Other	.01	.000	.000	.000
Age (Reference: ≥45 years)					
	18–29 years	−.07***	.000	−.01	−.01
	30–44 years	−.02*	−.004*	−.02***	−.02***
Education (Reference: High school)					
	8th grade	−.02	−.002	−.02**	−.01**
	CDA	−.01	.001	.003	.003
	Associate degree	.02**	−.002	.002	.000
	Bachelor’s degree	.13***	−.001	.01**	.01*
	Graduate degree	.10***	−.002	.01**	.01
Years as childcare professional (Reference: <6 years)					
	6–10 years	.01	−.001	.001	.000
	11–15 years	−.01	−.002	−.002	−.003
	>15 years	−.01	.000	.01**	.01*
Childcare setting (Reference: Commercial center)					
	Non-commercial	.06***	.001	.002	.002
	Religious	.07***	.002	.01***	.01***
	Head start	.01	.001	.003	.004
	Home based	.01	−.001	.000	−.001
	Other	.07***	.000	.01*	.01
Previously completed mandated reporter training (Reference: no prior training)					
		−.02*	−.01***	−.003	−.01***
Baseline knowledge		−.57***			
Acceptability		.08***			
Appropriateness		.14***			

Note: CDA = Child Development Associate credential. This table summarizes standardized coefficients for direct, indirect, and total effects reported in greater detail in Appendix Tables 2, 5, and 6. *p-value <.05; **p-value <0.01; ***p-value <0.001.

**Table 4.** Narrative summary by user characteristic of mediators of knowledge gains

		Mediation of knowledge gains narrative summary
Gender: Female		Females reported higher acceptability, which mediated knowledge gains
Race/ethnicity (Reference: White)		
	Asian	No evidence of mediation
	Hispanic	Hispanic users reported higher acceptability and appropriateness, which both mediated knowledge gains
	Black	Black users reported higher levels of appropriateness, which mediated knowledge outcomes
	Other	No evidence of mediation
Age (Reference: ≥45 years)		
	18–29 years	No evidence of mediation
	30–44 years	Users age 30–44 reported lower acceptability and appropriateness, both mediated knowledge gains
Education (Reference: High school) (highest level completed)		
	8th grade	Users with 8th grade education reported lower appropriateness, which mediated knowledge gains
	CDA	No evidence of mediation
	Associate degree	No evidence of mediation
	Bachelor’s degree	Users with a bachelor’s degree reported higher appropriateness, which mediated knowledge gains
	Graduate degree	Users with a graduate degree reported higher appropriateness, which mediated knowledge gains
Years as childcare professional (Reference: <6 years)		
	6–10 years	No evidence of mediation
	11–15 years	No evidence of mediation
	>15 years	Users with >15 years of experience reported higher appropriateness, which mediated knowledge gains
Childcare setting (Reference: Commercial center)		
	Non-commercial	No evidence of mediation
	Religious	Users from religious facilities reported higher appropriateness, which mediated knowledge gains
	Head start	No evidence of mediation
	Home based	No evidence of mediation
	Other	Users from "other" facilities reported higher appropriateness, which mediated knowledge gains
Previously completed mandated reporter training (Reference: no prior training)		Users with previous training reported lower acceptability and appropriateness, only acceptability mediated knowledge gains

### Race/ethnicity

Adjusting for baseline knowledge and other demographic variables, results of the SEM showed that, relative to White learners, Asian (*β* = −.06; 95%CI: −.08, −.05, *p* < .001), Hispanic (*β* = −.08; 95%CI: −.09, −.06, *p* < .001), and Black (*β* = −.16; 95%CI: −.17, −.14, *p* < .001) learners had less knowledge gains following the training (Appendix Table 2). Hispanic learners reported higher acceptability (Appendix Table 3); Asian, Hispanic, and Black learners reported higher appropriateness (compared to White learners; Appendix Table 4). For most, these ratings of the program’s appropriateness and acceptability translated into mediation via implementation outcomes, indicating that their rating of the *iLookOut* training’s acceptability and appropriateness did impact their knowledge change. Specifically, acceptability mediated knowledge gains for Hispanic learners (indirect effect: *β* = .01; 95%CI: .003, .01, *p* = 0.001), whereas appropriateness mediated knowledge gains for both Hispanic (indirect effect: *β* = .01; 95%CI: .003, .01, *p* = .001) and Black learners (indirect effect: *β* = .01; 95%CI: .003, .01, *p* = .002; Appendix Table 5). However, for both Hispanic and Black learners, a statistically significant direct effect remained. For Asian learners, neither acceptability nor appropriateness mediated the relationship with knowledge gains, after adjusting for implementation outcomes, a significant direct effect remained (Table 3).

### Gender

Gender was not associated with knowledge gains (Table 3). However, female learners reported higher acceptability of the training (*β* = .08; 95%CI: .05, .10, *p* < .001; Appendix Table 3), and the indirect effect via acceptability was statistically significant (Appendix Table 5). Although gender, per se, was not associated with knowledge gains, female learners gave (compared to males) higher acceptability ratings, which were associated with greater gains in knowledge.

### Age

Younger learners had lower knowledge gains (age 18–29: *β* = −.07, 95%CI: −.09, −.04, *p* < .001; age 30–44 years: *β* = −.02, 95%CI: −.04, −.001, *p* = .036) compared to learners ≥45 years old (Appendix Table 2). Acceptability (*β* = −.004, 95%CI: −.01, −.001, *p* = .014) and appropriateness (*β* = −.02, 95%CI: −.02, −.01, *p* < .001) emerged as mediators for knowledge change only for learners age 30–44 years (Appendix Table 5), where lower acceptability and appropriateness were associated with less knowledge gains. For the youngest learners (age 18–29 years), implementation outcomes did not explain associations with less knowledge gains, compared to older learners (≥45 years; Table 3).

### Education

Learners with higher levels of education had statistically significantly greater knowledge gains compared to those with a high school education (associate degree: *β* = .02, 95%CI: .01, .04, *p* = .002; bachelor’s degree: *β* = .13, 95%CI: .12, .15, *p* < .001; or graduate degree: *β* = .10, 95%CI: .08, .11, *p* < .001; Appendix Table 2). Acceptability did not mediate relationships between a learner’s education level and knowledge change (Table 3). Further, no mediation was found for learners with an associate degree, though a significant direct effect remained. Appropriateness mediated relationships between having a bachelor’s degree (*β* = .01, 95%CI: .01, .02, *p* = .001), a graduate degree (*β* = .01, 95%CI: .003, .02, *p* = .003), or an 8^th^ grade education (*β* = −.01, 95%CI: −.01, −.002, *p* = .004) and knowledge gains (Appendix Table 5). For these groups, finding the program to be more appropriate was associated with greater knowledge gains. A statistically significant direct relationship remained for learners with a bachelor’s or graduate degree and knowledge gains, indicating that program appropriateness did not fully explain post-training knowledge gain (Table 3).

### Years as practitioner

Number of years working as a childcare professional was not related to knowledge gains or acceptability (Table 3). However, those with more than 15 years of experience (compared to those with <6 years) found the training to be more appropriate (*β* = .05, 95%CI: .01, .08, *p* = .004; Appendix Table 4), and that appropriateness mediated the relationship with knowledge gains (Appendix Table 5). No mediation was found for learners with less than 15 years of childcare experience (Table 3).

### Childcare setting

Compared to those working in a commercial center, learners working in a non-commercial center (*β* = .06, 95%CI: .04, .08, *p* < 0.001), a religious center (*β* = .08, 95%CI: .06, .09, *p* < .001), or other type of childcare setting (*β* = .07, 95%CI: .05, .08, *p* < .001) had greater knowledge gains (Appendix Table 2). No association was found between knowledge gains and working in either a Head Start or home-based program (Table 3). Type of childcare setting was not associated with acceptability (Table 3); however, learners who worked in a religious facility (*β* = .06, 95%CI: .03, .09, *p* < .001) or “other setting” (*β* = .04, 95%CI: .004, .07, *p* = .040) reported higher appropriateness of the training (Appendix Table 4). Appropriateness partially mediated the associations between knowledge gains and religious facility or other setting. For learners working in a non-commercial setting, there was no statistically significant indirect effect via either implementation outcome (Table 3).

### Prior training

Prior mandated reporter training was associated with smaller gains in knowledge (compared to those without prior training; *β* = −.02, 95%CI: −.03, −.003, *p* = .015; Appendix Table 2). Those with prior training found the program to be less acceptable and less appropriate than those without (Table 3). Acceptability partially mediated the relationship between prior training and knowledge gain (direct effect: *β* = −.10, 95%CI: −.13, −.07, *p* < .001; indirect effect via acceptability: *β* = −.01, 95%CI: −.01, −.01, *p* < .001) such that among learners with prior mandated reporter training lower acceptability was associated with less knowledge gains (Appendix Tables 3 & 5).

## Discussion

This study found that learner demographic and professional characteristics were differentially associated with knowledge change, and that program components explained these differences for most groups. Certain implementation outcomes were strongly associated with knowledge gains, notably acceptability for female learners and appropriateness for learners who had not completed high school or had >15 years of experience in a childcare setting. So too, group membership maintained a statistically significant direct relationship with knowledge change pre- to post-training (specifically, for Asian, Hispanic, and Black learners, those 30–44 years of age, with a bachelor’s or graduate degree, those working in a religious facility, and those with prior mandated reporter training). Where mediation was found, for the majority of groups, appropriateness emerged as the driving mediator.

Previous studies examining efficacy of the *iLookOut* Core Training have found strong evidence that *iLookOut* outperformed a standard mandated reporter training in a randomized controlled trial [13,15] and resulted in knowledge gains in a real-world setting [16]. Additionally, the real-world setting study found that learner age, race, and education were statistically significant predictors of knowledge change following the training. The present study aimed to build upon these results by examining whether (and how) knowledge change was associated with implementation outcomes. Building on Proctor’s conceptual framework [18] for evaluating program implementation, we investigated whether implementation outcomes, specifically acceptability and appropriateness differentially mediated knowledge gains for specific groups – that is, was program acceptability or appropriateness responsible for differences in knowledge gain by learner characteristic.

Our finding that implementation outcomes are related to efficacy of a training on the sensitive topic of child maltreatment is consistent with other studies examining the relationship between acceptability and effectiveness [37–39], as well as higher satisfaction with increases in knowledge 40. However, few studies have considered the impact of end-user demographics or professional characteristics on implementation outcomes and efficacy [18,28].

Amidst the many associations identified, we believe the most salient findings involve the relationship between specific program components and characteristics of learners for whom the training was most (or least) effective. For example, learners who had not graduated high school demonstrated among the lowest knowledge gains despite having low baseline knowledge scores. As with prior research on the *iLookOut* training [15] and other interventions [39], lower baseline knowledge scores emerged as the strongest overall predictor of greater knowledge gains. This is important since it indicates that ECPs who are most in need of training benefit the most from the *iLookOut* training. Although, overall, learners without a high school education had lower knowledge gains compared to those with a high school education, those without a high school education did, as a group, endorse program components as appropriate at higher rates than those who graduated high school. Further, appropriateness emerged as a mediator for knowledge gains for those without a high school education, indicating that for those who did find the program appropriate, this was related to knowledge gain. Black learners had the lowest mean knowledge gain; however, there was not a clear pattern based on endorsement of item-level indicators of appropriateness, e.g., they endorsed program aspects such as reporting procedures or online availability at similar rates as other racial or ethnic groups. Although similar to learners without a high school education, appropriateness of the program emerged as a mediator for knowledge gains. More work is needed to understand what may be driving lower overall knowledge gains among Black learners. Lastly, this study did not investigate why certain program components were rated more or less appropriate by specific user groups, additional investigation is needed to understand how the training can be altered to increase knowledge gains for these groups.

Consistent with other studies of web-based learning [41], learners with a bachelor’s or graduate degree experienced the largest knowledge gains. These groups were also significantly more likely to endorse the value of resource documents and explanation of legal requirements, with item-level indicators of appropriateness mediating their higher knowledge gains.

Additionally important is understanding how group membership may overlap to impact training outcomes. Importantly, several user characteristics evaluated may represent causal relationships with knowledge gains (e.g., education level likely impacts a user’s ability to understand more complex content or number of years teaching may drive relevance or engagement with the *iLookOut* training). In the present study, race or ethnicity was included as a covariate based on previous findings that knowledge gain differed significantly by racial group.

However, race or ethnicity was not hypothesized here to be causal for knowledge outcomes; we acknowledge that differences seen are likely related to unmeasured racialized structures. Findings from this study show that Asian, Hispanic, and Black learners had statistically significantly lower knowledge gains than White learners. Implementation outcomes may help explain part of this, further, for Asian, Hispanic, and Black learners, some unmeasured learner characteristics may have also contributed to these lesser gains. For example, other studies have found that learner age [21] and education level [41] impact training outcomes. In the present study, we adjusted for these factors in analyses; however, there are likely important unmeasured characteristics of learners or factors associated with racialized structures that were not accounted for and may be contributing to learner discrepancies by racial/ethnic group. Further, Asian and Hispanic learners were less likely to have had previous mandated reporter training compared to White learners. We would expect not having previous training to lead to greater knowledge gains given that there is likely more to learn about child maltreatment signs and reporting procedures. Given that appropriateness partially mediated knowledge change in these groups, appropriateness of the program was important for learner outcomes, but other factors related to underrepresented racial/ethnic groups also appear to be driving knowledge change. Subsequent analyses should aim to better understand drivers of this relationship to identify how these lesser knowledge gains may be related to other structural factors, demographic, or professional characteristics. This may provide guidance on tailoring program components to increase knowledge gains for these groups e.g., to understand how multiple learner characteristics may interact to impact perceived usefulness of the program, and ultimately, knowledge change.

## Strengths and limitations

Strengths of the present study include its large sample size, formal evaluation of acceptability and appropriateness and their impact on knowledge change, and use of a real-world setting. However, the study was not designed to assess various other implementation outcomes, including feasibility, adoption, fidelity, and penetration [18]. Moreover, while our study provides information about which implementation outcomes impact knowledge change for specific groups, and which program components are most impactful, the present findings do not explain *why* specific components are less effective for certain groups, nor what changes are needed to increase knowledge gains for these groups. For such an understanding, in-depth qualitative work is needed. That said, the present findings reinforce the critical role that implementation outcomes have for ensuring that interventions are effective [26,27].

## Conclusion

Implementation outcomes emerged as important drivers of knowledge change for multiple groups. *Appropriateness* emerged as a key mediator, providing support that *iLookOut’s* content and approach are important for knowledge change for a majority of learners. Moreover, the *iLookOut* Core Training’s use of a multimedia learning environment, video-based storylines, and game-based techniques [22,23] were both endorsed by learners and correlated with increases in knowledge about child maltreatment and its reporting. Future work could build upon these findings by exploring first, *why* various aspects of the *iLookOut* training are rated by some groups as less acceptable or appropriate, and second, what changes would improve efficacy for low performing learners. Learner differences in acceptability and appropriateness of the *iLookOut* training may relate to how learners’ experiences or background impacts their engagement with the training, e.g., their age, cultural background, professional duties, or experience with technology may impact their training experience. Several studies have found that younger learners experience fewer technical difficulties with online trainings, whereas low-income learners experience greater technical difficulties [42]. Others have found that feelings of competency and a sense of affiliation with the training material are important drivers of learner outcomes [43]. For example, some users may identify more with vignettes presented based on the specific story or race or ethnicity of characters, impacting their engagement and learning. Users who perceive the training to be more relevant to their role as mandated reporters, possibly based on having made prior reports, or based on their professional designation (e.g., full-time versus volunteer) may be more invested in the training [44]. Future research should aim to better understand the reasons *why* specific user groups rated program aspects differently. Because web-based trainings are more easily tailored than many other training modalities, there are significant advantages to understanding what aspects of an online training optimize outcomes, and for whom. As such, the present findings can help inform the development of other web-based trainings with respect to content, approach, and which program components may help improve outcomes for specific learners.

## Supporting information

Barnett et al. supplementary material Barnett et al. supplementary material

## Supplementary material

The supplementary material for this article can be found at https://doi.org/10.1017/cts.2023.628.
