# Supplementary material for: Identifying what works for whom: Implementation outcomes following iLookOut, a child abuse identification and referral training program
Source: J Clin Transl Sci. 2023 Sep 15;7(1):e205. doi: 10.1017/cts.2023.628 (PMC10565203; doi:10.1017/cts.2023.628)
Supplement: Barnett et al. supplementary material [file S2059866123006283sup001.docx]

**Appendix Tables**

Identifying what works for whom: Implementation outcomes following *iLookOut*, a child abuse identification and referral training program.

**Appendix Table 1.** Baseline and post-training knowledge scores and knowledge change

(pre- to post-training) by learner characteristic

|  |  | Baseline knowledge score | Post-training knowledge score | Knowledge change |
| --- | --- | --- | --- | --- |
|  |  | mean (S.D.) | mean (S.D.) | mean (S.D.) |
| Gender | |  |  |  |
|  | Female | 13.8 (2.9) | 16.9 (2.9) | 3.1 (3.2) |
|  | Male | 13.8 (3.0) | 17.0 (3.0) | 3.2 (3.4) |
| Race/ethnicity | |  |  |  |
|  | White | 14.0 (2.9) | 17.4 (2.8) | 3.4 (3.2) |
|  | Asian | 13.3 (3.3) | 16.1 (3.2) | 2.8 (3.2) |
|  | Hispanic | 13.3 (2.9) | 16.0 (2.9) | 2.7 (3.0) |
|  | Black | 13.5 (2.8) | 15.8 (2.9) | 2.3 (2.9) |
|  | Other | 13.2 (3.3) | 16.4 (3.3) | 3.2 (3.4) |
| Age | |  |  |  |
|  | > 45 years | 13.9 (3.1) | 17.4 (2.8) | 3.5 (3.3) |
|  | 18-29 years | 13.6 (2.7) | 16.6 (3.0) | 2.9 (3.1) |
|  | 30-44 years | 14.1 (3.0) | 17.2 (2.9) | 3.0 (3.2) |
| Education (highest level completed) | |  |  |  |
|  | High school | 13.4 (2.8) | 16.3 (3.0) | 2.9 (3.1) |
|  | 8th grade | 12.7 (3.4) | 15.2 (3.2) | 2.5 (3.6) |
|  | CDA | 13.9 (3.1) | 16.3 (2.9) | 2.4 (3.1) |
|  | Associate degree | 13.9 (2.9) | 16.8 (2.7) | 2.9 (3.0) |
|  | Bachelor’s degree | 14.2 (2.9) | 17.8 (2.7) | 3.6 (3.3) |
|  | Graduate degree | 14.5 (3.0) | 18.1 (2.6) | 3.6 (3.2) |
| Years as childcare professional | |  |  |  |
|  | < 6 years | 13.7 (2.8) | 16.8 (3.0) | 3.1 (3.2) |
|  | 6-10 years | 14.0 (3.0) | 17.1 (2.8) | 3.1 (3.2) |
|  | 11-15 years | 14.0 (3.1) | 17.0 (2.9) | 3.0 (3.2) |
|  | >15 years | 14.3 (3.0) | 17.5 (2.8) | 3.1 (3.2) |
| Childcare setting | |  |  |  |
|  | Commercial | 13.6 (2.8) | 16.3 (2.9) | 2.8 (3.0) |
|  | Non-commercial | 13.8 (2.9) | 16.9 (2.9) | 3.1 (3.1) |
|  | Religious | 13.5 (3.0) | 17.6 (2.9) | 4.0 (3.4) |
|  | Head start | 14.1 (2.8) | 16.8 (2.9) | 2.7 (3.0) |
|  | Home based | 13.9 (3.1) | 16.6 (3.1) | 2.7 (3.1) |
|  | Other | 14.2 (2.9) | 17.7 (2.7) | 3.5 (3.3) |
| Prior training | |  |  |  |
|  | Yes | 14.2 (2.8) | 17.1 (2.9) | 2.9 (3.0) |
|  | No | 13.1 (2.9) | 16.5 (3.0) | 3.5 (3.4) |

Note: CDA = Child Development Associate credential

**Appendix Table 2.** Unstandardized and standardized path coefficients, indirect effects, and bootstrapped confidence intervals (direct effects to knowledge change)

| Path | |  | | | | | | |  | | | **95% CI** | | | |
| --- | --- | --- | --- | --- | --- | --- | --- | --- | --- | --- | --- | --- | --- | --- | --- |
| Direct effects to knowledge change | | | | **Coef** | | **SE** | | **β** | | | **p-value** | | | **LL** | **UL** |
| Gender: Female | | | -.06 | | .08 | | -.01 | | | .470 | | | -.019 | | .01 |
| Race/ethnicity (Reference: White) | | |  | |  | |  | | |  | | |  | |  |
|  | Asian | | -1.37 | | .18 | | -.06 | | | <.001 | | | -.08 | | -.05 |
|  | Hispanic | | -1.02 | | .10 | | -.08 | | | <.001 | | | -.09 | | -.06 |
|  | Black | | -1.22 | | .06 | | -.16 | | | <.001 | | | -.17 | | -.14 |
|  | Other | | .17 | | .15 | | .01 | | | .250 | | | -.00 | | .02 |
| Age (Reference: >45 years) | | |  | |  | |  | | |  | | |  | |  |
|  | 18-29 years | | -.42 | | .07 | | -.07 | | | <.001 | | | -.09 | | -.04 |
|  | 30-44 years | | -.14 | | .07 | | -.02 | | | .036 | | | -.04 | | -.001 |
| Education (Reference: High school) (highest level completed) | | |  | |  | |  | | |  | | |  | |  |
|  | 8th grade | | -.55 | | .32 | | -.02 | | | .086 | | | -.03 | | .003 |
|  | CDA | | -.06 | | .10 | | -.01 | | | .524 | | | -.02 | | .01 |
|  | Associate degree | | .23 | | .07 | | .02 | | | .002 | | | .01 | | .04 |
|  | Bachelor’s degree | | .95 | | .07 | | .13 | | | <.001 | | | .12 | | .15 |
|  | Graduate degree | | .99 | | .09 | | .10 | | | <.001 | | | .08 | | .11 |
| Years as childcare professional  (Reference: <6 years) | | | | |  | |  | | |  | | |  | |  |
|  | 6-10 years | | .05 | | .07 | | .01 | | | .497 | | | -.01 | | .02 |
|  | 11-15 years | | -.08 | | .09 | | -.01 | | | .418 | | | -.02 | | .01 |
|  | >15 years | | -.11 | | .08 | | -.01 | | | .166 | | | -.03 | | .01 |
| Childcare setting (Reference: Commercial center) | | | | |  | |  | | |  | | |  | |  |
|  | Non-commercial | | .41 | | .06 | | .06 | | | <.001 | | | .04 | | .08 |
|  | Religious | | .82 | | .09 | | .08 | | | <.001 | | | .06 | | .09 |
|  | Head start | | .07 | | .09 | | .01 | | | .447 | | | -.01 | | .02 |
|  | Home based | | .15 | | .11 | | .01 | | | .174 | | | -.01 | | .03 |
|  | Other | | .56 | | .08 | | .07 | | | <.001 | | | .05 | | .08 |
| Previously completed mandated reporter training  (Reference: no prior training) | | | -.13 | | .05 | | -.02 | | | .015 | | | -.03 | | -.003 |
| Baseline knowledge | | | -.62 | | .01 | | -.57 | | | <.001 | | | -.59 | | -.56 |
| Acceptability | | | .22 | | .03 | | .08 | | | <.001 | | | .06 | | .11 |
| Appropriateness | | | .75 | | .07 | | .14 | | | <.001 | | | .11 | | .16 |

Note: CDA = Child Development Associate credential

**Appendix Table 3.** Unstandardized and standardized path coefficients, indirect effects, and bootstrapped confidence intervals (direct effects to acceptability)

| Path | |  |  |  |  | **95% CL** | |
| --- | --- | --- | --- | --- | --- | --- | --- |
| Direct effects to acceptability | | **Coef** | **SE** | **β** | **p-value** | **LL** | **UL** |
| Gender: Female | | .29 | .06 | .08 | <.001 | .05 | .10 |
| Race/ethnicity (Reference: White) | |  |  |  |  |  |  |
|  | Asian | .18 | .14 | .02 | .169 | -.01 | .05 |
|  | Hispanic | .30 | .08 | .06 | <.001 | .03 | .09 |
|  | Black | .08 | .05 | .03 | .085 | -.01 | .06 |
|  | Other | .01 | .12 | .001 | .949 | -.03 | .03 |
| Age (Reference: >45 years) | |  |  |  |  |  |  |
|  | 18-29 years | -.01 | .06 | -.004 | .864 | -.05 | .05 |
|  | 30-44 years | -.14 | .05 | -.05 | .007 | -.09 | -.01 |
| Education (Reference: High school) (highest level completed) | |  |  |  |  |  |  |
|  | 8th grade | -.29 | .20 | -.02 | .138 | -.05 | .01 |
|  | CDA | .03 | .08 | .01 | .677 | -.02 | .04 |
|  | Associate degree | -.09 | .05 | -.03 | .108 | -.05 | .01 |
|  | Bachelor’s degree | -.04 | .05 | -.02 | .355 | -.05 | .02 |
|  | Graduate degree | -.10 | .07 | -.03 | .173 | -.06 | .01 |
| Years as childcare professional  (Reference: <6 years) | | |  |  |  |  |  |
|  | 6-10 years | -.05 | .05 | -.02 | .328 | -.04 | .02 |
|  | 11-15 years | -.09 | .07 | -.02 | .174 | -.05 | .01 |
|  | >15 years | .01 | .06 | .001 | .940 | -.03 | .04 |
| Childcare setting (Reference: Commercial center) | |  |  |  |  |  |  |
|  | Non-commercial | .03 | .05 | .01 | .585 | -.03 | .04 |
|  | Religious | .10 | .07 | .02 | .154 | -.01 | .06 |
|  | Head start | .04 | .07 | .01 | .597 | -.02 | .04 |
|  | Home based | -.05 | .08 | -.01 | .564 | -.04 | .02 |
|  | Other | -.01 | .06 | -.003 | .888 | -.04 | .03 |
| Previously completed mandated reporter training (Reference: no prior training) | | -.25 | .04 | -.10 | <.001 | -.13 | -.07 |

Note: CDA = Child Development Associate credential

**Appendix Table 4.** Unstandardized and standardized path coefficients, indirect effects, and bootstrapped confidence intervals (direct effects to appropriateness)

| Path | |  |  |  |  | **95% CL** | |
| --- | --- | --- | --- | --- | --- | --- | --- |
| Direct effects to appropriateness | | **Coef** | **SE** | **β** | **p-value** | **LL** | **UL** |
| Gender: Female | | .06 | .03 | .03 | .064 | -.004 | .06 |
| Race/ethnicity (Reference: White) | |  |  |  |  |  |  |
|  | Asian | .11 | .05 | .03 | .048 | <.001 | .05 |
|  | Hispanic | .12 | .04 | .05 | <.001 | .02 | .08 |
|  | Black | .07 | .02 | .05 | .001 | .02 | .07 |
|  | Other | -.003 | .05 | -.001 | .948 | -.03 | .02 |
| Age (Reference: >45 years) | |  |  |  |  |  |  |
|  | 18-29 years | -.05 | .03 | -.04 | .106 | -.09 | .01 |
|  | 30-44 years | -.14 | .02 | -.11 | <.001 | -.14 | -.07 |
| Education (Reference: High school) (highest level completed) | |  |  |  |  |  |  |
|  | 8th grade | -.28 | .09 | -.04 | .002 | -.07 | -.02 |
|  | CDA | .04 | .03 | .02 | .177 | -.01 | .05 |
|  | Associate degree | .03 | .03 | .02 | .287 | -.01 | .05 |
|  | Bachelor’s degree | .10 | .03 | .08 | <.001 | .04 | .12 |
|  | Graduate degree | .13 | .04 | .07 | .002 | .02 | .11 |
| Years as childcare professional  (Reference: <6 years) | | |  |  |  |  |  |
|  | 6-10 years | .01 | .02 | .01 | .559 | -.02 | .04 |
|  | 11-15 years | -.02 | .03 | -.01 | .426 | -.04 | .01 |
|  | >15 years | .08 | .03 | .05 | .004 | .01 | .08 |
| Childcare setting (Reference: Commercial center) | |  |  |  |  |  |  |
|  | Non-commercial | .01 | .02 | .01 | .482 | -.02 | .04 |
|  | Religious | .12 | .03 | .06 | <.001 | .03 | .09 |
|  | Head start | .05 | .03 | .02 | .101 | -.002 | .05 |
|  | Home based | .003 | .04 | .001 | .940 | -.03 | .03 |
|  | Other | .06 | .03 | .04 | .040 | .004 | .07 |
| Previously completed mandated reporter training (Reference: no prior training) | | -.03 | .02 | -.02 | .040 | -.05 | .01 |

Note: CDA = Child Development Associate credential

**Appendix Table 5.** Unstandardized and standardized path coefficients, indirect effects, and bootstrapped confidence intervals (indirect effects via acceptability and appropriateness)

|  | | **Indirect effects via acceptability** | | | | | | | | | | | **Indirect effects via appropriateness** | | | | | | | | | | | | | | |
| --- | --- | --- | --- | --- | --- | --- | --- | --- | --- | --- | --- | --- | --- | --- | --- | --- | --- | --- | --- | --- | --- | --- | --- | --- | --- | --- | --- |
| Path | |  |  | |  | |  | | **95% CL** | | | |  | |  | |  | |  | | | **95% CL** | | | | | |
|  | | **Coef** | **SE** | | **β** | | **p-value** | | **LL** | | **UL** | | **Coef** | | **SE** | | **β** | | **p-value** | | | **LL** | | | **UL** | | |
| Gender: Female | | .07 | .02 | | .01 | | <.001 | | .004 | | .01 | | .05 | | .03 | | .004 | | .071 | | | -.001 | | | .01 | | |
| Race/ethnicity (Reference: White) | |  |  | |  | |  | |  | |  | |  | |  | |  | |  | | |  | | |  | | |
|  | Asian | .04 | .03 | | .002 | | .184 | | -.001 | | .01 | | .08 | | .04 | | .004 | | .052 | | | <.001 | | | .01 | | |
|  | Hispanic | .07 | .02 | | .01 | | .001 | | .003 | | .01 | | .09 | | .03 | | .01 | | .001 | | | .003 | | | .01 | | |
|  | Black | .02 | .01 | | .002 | | .093 | | <.001 | | .01 | | .05 | | .02 | | .01 | | .002 | | | .003 | | | .01 | | |
|  | Other | .002 | .03 | | .00 | | .951 | | -.003 | | .003 | | -.002 | | .04 | | .00 | | .950 | | | -.004 | | | .003 | | |
| Age (Reference: >45 years) | |  |  | |  | |  | |  | |  | |  | |  | |  | |  | | |  | | |  | | |
|  | 18-29 years | -.003 | .01 | | .00 | | .850 | | .01 | | .004 | | -.04 | | .02 | | -.01 | | .117 | | | -.02 | | | .001 | | |
|  | 30-44 years | -.03 | .01 | | -.004 | | .014 | | -.01 | | -.001 | | -.11 | | .02 | | -.02 | | <.001 | | | -.02 | | | -.01 | | |
| Education (Reference: High school) (highest level completed) | |  |  | |  | |  | |  | |  | |  | |  | |  | |  | | |  | | |  | | |
|  | 8th grade | -.07 | .05 | | -.002 | | .149 | | -.01 | | <.001 | | -.21 | | .07 | | -.01 | | .004 | | | -.01 | | | -.002 | | |
|  | CDA | .01 | .02 | | .001 | | .680 | | -.002 | | .003 | | .03 | | .03 | | .003 | | .184 | | | -.001 | | | .01 | | |
|  | Associate degree | -.02 | .01 | | -.002 | | .127 | | -.01 | | <.001 | | .02 | | .02 | | .002 | | .289 | | | -.002 | | | .01 | | |
|  | Bachelor’s degree | -.01 | .01 | | -.001 | | .373 | | -.01 | | .001 | | .08 | | .03 | | .01 | | .001 | | | .01 | | | .02 | | |
|  | Graduate degree | -.02 | .02 | | -.002 | | .196 | | -.01 | | .001 | | .10 | | .03 | | .01 | | .003 | | | .003 | | | .02 | | |
| Years as childcare professional | |  | |  | |  | |  | |  | |  | |  | |  | |  | |  | | |  | | |  |  |
| (Reference: <6 years) | |  |  |  |  |  |  |  |  |  |  |  | |  |  |  |  |  |  |  |  |  |  |  |  |  |  |
|  | 6-10 years | -.01 | .01 | | -.001 | | .337 | | -.004 | | .002 | | .01 | | .02 | | .001 | | .561 | | -.003 | | | .01 | | |  |
|  | 11-15 years | -.02 | .02 | | -.002 | | .194 | | -.01 | | .001 | | -.02 | | .02 | | -.002 | | .427 | | -.01 | | | .002 | | |  |
|  | >15 years | .001 | .01 | | .00 | | .937 | | -.003 | | .003 | | .06 | | .02 | | .01 | | .005 | | .002 | | | .01 | | |  |
| Childcare setting (Reference: Commercial center) | |  |  | |  | |  | |  | |  | |  | |  | |  | |  | |  | | |  | | |  |
|  | Non-commercial | .01 | .01 | | .001 | | .590 | | -.003 | | .01 | | .01 | | .02 | | .002 | | .485 | | -.003 | | | .01 | | |  |
|  | Religious | .02 | .02 | | .002 | | .162 | | -.001 | | .01 | | .09 | | .02 | | .01 | | <.001 | | .004 | | | .01 | | |  |
|  | Head start | .01 | .02 | | .001 | | .596 | | -.002 | | .004 | | .04 | | .02 | | .003 | | .105 | | <.001 | | | .01 | | |  |
|  | Home based | -.01 | .02 | | -.001 | | .572 | | -.004 | | .002 | | .002 | | .03 | | .00 | | .940 | | -.004 | | | .01 | | |  |
|  | Other | -.01 | .01 | | .00 | | .889 | | -.004 | | .003 | | .04 | | .02 | | .01 | | .044 | | <.001 | | | .01 | | |  |
| Previously completed mandated reporter training (Reference: no prior training) | | -.06 | .01 | | -.01 | | <.001 | | -.01 | | -.01 | | -.02 | | .02 | | -.003 | | .139 | | -.01 | | | .001 | | |  |

Note: CDA = Child Development Associate credential

**Appendix Table 6.** Unstandardized and standardized path coefficients, indirect effects, and bootstrapped confidence intervals (total effects)

|  | |  |  |  |  | **95% CL** | |
| --- | --- | --- | --- | --- | --- | --- | --- |
| Total effects | | **Coef** | **SE** | **β** | **p-value** | **LL** | **UL** |
| Gender: Female | | .05 | .08 | .01 | .508 | -.01 | .02 |
| Race/ethnicity (Reference: White) | |  |  |  |  |  |  |
|  | Asian | -1.25 | .17 | -.06 | <.001 | -.08 | -.045 |
|  | Hispanic | -.86 | .10 | -.07 | <.001 | -.09 | -.06 |
|  | Black | -1.15 | .06 | -.15 | <.001 | -.18 | -.14 |
|  | Other | .17 | .14 | .01 | .247 | -.01 | .03 |
| Age (Reference: >45 years) | |  |  |  |  |  |  |
|  | 18-29 years | -.46 | .07 | -.07 | <.001 | -.10 | -.05 |
|  | 30-44 years | -.28 | .07 | -.04 | <.001 | -.07 | -.02 |
| Education (Reference: High school) (highest level completed) | |  |  |  |  |  |  |
|  | 8th grade | -.82 | .30 | -.02 | .007 | -.04 | -.01 |
|  | CDA | -.02 | .10 | -.002 | .813 | -.02 | .01 |
|  | Associate degree | .23 | .07 | .03 | .001 | .01 | .04 |
|  | Bachelor’s degree | 1.02 | .06 | .14 | <.001 | .14 | .17 |
|  | Graduate degree | 1.06 | .09 | .10 | <.001 | .09 | .13 |
| Years as childcare professional  (Reference: <6 years) | | |  |  |  |  |  |
|  | 6-10 years | .05 | .07 | .01 | .505 | -.01 | .02 |
|  | 11-15 years | -.11 | .10 | -.01 | .234 | -.03 | .01 |
|  | >15 years | -.05 | .08 | -.01 | .523 | .00 | .01 |
| Childcare setting (Reference: Commercial center) | |  |  |  |  |  |  |
|  | Non-commercial | .43 | .06 | .06 | <.001 | .05 | .09 |
|  | Religious | .93 | .09 | .09 | <.001 | .07 | .11 |
|  | Head start | .12 | .09 | .01 | .204 | -.01 | .03 |
|  | Home based | .14 | .11 | .01 | .202 | -.01 | .03 |
|  | Other | .40 | .08 | .07 | <.001 | .06 | .10 |
| Previously completed mandated reporter training (Reference: no prior training) | | -.21 | .05 | -.03 | <.001 | -.05 | -.02 |

Note: CDA = Child Development Associate credential

**Appendix Table 7.** Unstandardized and standardized path coefficients, indirect effects, and bootstrapped confidence intervals (total indirect effects)

|  | |  |  |  |  | **95% CL** | |
| --- | --- | --- | --- | --- | --- | --- | --- |
| Total indirect effects | | **Coef** | **SE** | **β** | **p-value** | **LL** | **UL** |
| Gender: Female | | .11 | .03 | .01 | <.001 | .01 | .02 |
| Race/ethnicity (Reference: White) | |  |  |  |  |  |  |
|  | Asian | .12 | .06 | .01 | .026 | .001 | .01 |
|  | Hispanic | .16 | .04 | .01 | <.001 | .01 | .02 |
|  | Black | .07 | .02 | .01 | .001 | .004 | .02 |
|  | Other | -.001 | .05 | .00 | .991 | -.01 | .01 |
| Age (Reference: > 45 years) | |  |  |  |  |  |  |
|  | 18-29 years | -.04 | .03 | -.01 | .149 | -.02 | .002 |
|  | 30-44 years | -.14 | .02 | -.02 | <.001 | -.03 | -.01 |
| Education (Reference: High school) (highest level completed) | |  |  |  |  |  |  |
|  | 8th grade | -.28 | .07 | -.01 | .001 | -.01 | -.004 |
|  | CDA | .04 | .03 | .003 | .207 | -.002 | .01 |
|  | Associate degree | .004 | .03 | .00 | .884 | -.01 | .01 |
|  | Bachelor’s degree | .07 | .03 | .01 | .010 | .002 | .02 |
|  | Graduate degree | .07 | .04 | .01 | .051 | <.001 | .02 |
| Years as childcare professional  (Reference: <6 years) | | |  |  |  |  |  |
|  | 6-10 years | -.001 | .02 | .00 | .977 | -.01 | .01 |
|  | 11-15 years | -.04 | .03 | -.003 | .179 | -.01 | .001 |
|  | >15 years | .06 | .03 | .01 | .018 | .001 | .01 |
| Childcare setting (Reference: Commercial center) | |  |  |  |  |  |  |
|  | Non-commercial | .02 | .02 | .002 | .394 | -.003 | .01 |
|  | Religious | .11 | .03 | .01 | <.001 | .004 | .02 |
|  | Head start | .05 | .03 | .004 | .118 | -.001 | .01 |
|  | Home based | -.01 | .04 | -.001 | .820 | -.01 | .01 |
|  | Other | .04 | .03 | .01 | .118 | -.002 | .01 |
| Previously completed mandated reporter training (Reference: no prior training) | | -.08 | .02 | -.01 | <.001 | -.02 | -.01 |

Note: CDA = Child Development Associate credential

**Appendix Table 8.** Early Childhood Professional (ECP) associated with race or ethnicity

|  |  | Race/ethnicity | | | | |
| --- | --- | --- | --- | --- | --- | --- |
|  |  | White | Asian | Hispanic | Black | Other |
|  |  | χ^2^ (4) = 46.22, p-value<.001 | | | | |
| Gender: Female | | 90.0 | 82.1 | 90.5 | 86.4 | 85.3 |
| Age | | X2 (8) = 111.9 | | | | |
|  | >45 years | 27.2 | 17.9 | 14.3 | 23.0 | 16.4 |
|  | 18-29 years | 47.2 | 44.2 | 59.0 | 50.2 | 57.8 |
|  | 30-44 years | 25.6 | 38.0 | 26.7 | 26.9 | 25.8 |
| Education (highest level completed) | | χ^2^ (20) = 630.1, p-value<.001 | | | | |
|  | High school | 39.4 | 26.3 | 52 | 50.8 | 40.2 |
|  | 8th grade | 0.7 | 0.0 | 1.6 | 1.0 | 0.8 |
|  | CDA | 5.0 | 2.6 | 9.1 | 11.0 | 7.4 |
|  | Associate degree | 12.6 | 7.3 | 16.3 | 14.5 | 16.4 |
|  | Bachelor’s degree | 30.5 | 16.4 | 17.2 | 15.5 | 24.6 |
|  | Graduate degree | 11.9 | 29.9 | 3.9 | 7.0 | 10.7 |
| Years as childcare professional | | χ^2^ (12) = 93.4, p-value<.001 | | | | |
|  | < 6 years | 63.6 | 72.3 | 74.2 | 62.1 | 69.3 |
|  | 6-10 years | 13.8 | 13.5 | 13.8 | 16.9 | 14.3 |
|  | 11-15 years | 7.6 | 5.8 | 6.3 | 7.9 | 7.4 |
|  | >15 years | 15.0 | 7.7 | 5.7 | 13.1 | 9.0 |
| Childcare setting | | χ^2^ (20) = 285.9, p-value<.001 | | | | |
|  | Commercial | 23.5 | 22.3 | 30.7 | 27.9 | 27.1 |
|  | Non-commercial | 34.8 | 29.9 | 33.6 | 40.9 | 33.6 |
|  | Religious | 11.2 | 9.5 | 7.4 | 6.0 | 6.6 |
|  | Head start | 7.8 | 7.7 | 7.9 | 7.5 | 7.8 |
|  | Home based | 4.5 | 3.3 | 6.6 | 8.2 | 5.7 |
|  | Other | 18.2 | 27.4 | 13.7 | 9.6 | 19.3 |
| Previously completed mandated reporter training | | χ^2^ (4) = 115.4, p-value<.001 | | | | |
|  | Yes | 69.0 | 50.7 | 55.2 | 62.7 | 63.1 |

Note: CDA = Child Development Associate credential

**Appendix Table 9**. Item level indicators of acceptability by learner characteristic

|  | | Ease of learning from information | | | Program kept my interest & attention | | | Information was useful for my role as a mandated reporter | | | Information was provided in a way that helped me learn | | | Storyline was helpful for learning | | | Interactive scenarios | Videos |
| --- | --- | --- | --- | --- | --- | --- | --- | --- | --- | --- | --- | --- | --- | --- | --- | --- | --- | --- |
|  |  | Disagree | Neutral | Agree | Disagree | Neutral | Agree | Disagree | Neutral | Agree | Disagree | Neutral | Agree | Disagree | Neutral | Agree | Yes | Yes |
| Gender | | χ^2^ (2) = 0.85 | | | χ^2^ (2) = 11.87** | | | χ^2^ (2) = 20.24*** | | | χ^2^ (2) = 18.09*** | | | χ^2^ (2) = 30.67*** | | | χ^2^ (2) = 18.15*** | χ^2^ (2)  = 0.69 |
|  | Female | 3.3 | 9.3 | 87.4 | 2.6 | 8.2 | 89.2 | 0.8 | 3.7 | 95.4 | 1.1 | 4.5 | 94.5 | 1.1 | 4.1 | 94.8 | 51.2 | 55.4 |
|  | Male | 2.9 | 9.5 | 87.6 | 3.5 | 10.3 | 86.2 | 1.0 | 6.2 | 92.8 | 1.9 | 6.4 | 91.7 | 1.9 | 6.8 | 91.2 | 45.2 | 54.2 |
| Race/ethnicity | | χ^2^ (2) = 95.72*** | | | χ^2^ (2) = 33.79*** | | | χ^2^ (2) = 16.76* | | | χ^2^ (2) = 26.66** | | | χ^2^ (2) = 29.93*** | | | χ^2^ (2) =17.12** | χ^2^ (2)  = 147.13*** |
|  | White | 3.1 | 8.0 | 88.9 | 2.7 | 8.8 | 88.6 | 0.8 | 4.1 | 95.1 | 1.2 | 4.9 | 93.9 | 1.2 | 4.5 | 94.3 | 51.8 | 51.9 |
|  | Asian | 1.8 | 7.7 | 90.4 | 2.6 | 7.4 | 90.0 | 0.4 | 2.6 | 97.1 | 0.7 | 2.9 | 96.3 | 1.1 | 3.7 | 95.2 | 49.7 | 58.8 |
|  | Hispanic | 1.2 | 10.2 | 88.6 | 1.4 | 5.6 | 92.9 | 0.7 | 2.5 | 96.9 | 0.7 | 2.4 | 97.0 | 0.4 | 3.2 | 96.5 | 47.3 | 63.2 |
|  | Black | 4.2 | 13.1 | 82.7 | 2.8 | 7.7 | 89.7 | 0.9 | 3.9 | 95.3 | 0.9 | 4.5 | 94.7 | 1.1 | 3.4 | 94.9 | 47.9 | 64.2 |
|  | Other | 6.1 | 12.3 | 81.6 | 5.8 | 13.2 | 81.1 | 1.7 | 7.4 | 91.0 | 2.1 | 8.7 | 89.3 | 2.5 | 9.8 | 87.7 | 48.0 | 51.6 |
| Age | | χ^2^ (2) = 70.69*** | | | χ^2^ (2) = 36.67*** | | | χ^2^ (2) = 11.16* | | | χ^2^ (2) = 9.44 | | | χ^2^ (2) = 5.15 | | | χ^2^ (2) = 16.19*** | χ^2^ (2)  = 35.94*** |
|  | > 45 years | 5.0 | 11.3 | 83.7 | 2.2 | 6.5 | 91.4 | 0.8 | 3.6 | 95.7 | 1.1 | 4.5 | 94.4 | 1.2 | 4.2 | 94.6 | 49.1 | 54.6 |
|  | 18-29 years | 2.5 | 8.2 | 89.4 | 3.2 | 9.5 | 87.4 | 0.8 | 3.8 | 95.5 | 1.2 | 4.3 | 94.5 | 1.2 | 4.1 | 94.7 | 52.4 | 57.7 |
|  | 30-44 years | 3.1 | 9.5 | 87.4 | 2.3 | 8.3 | 89.4 | 1.1 | 4.8 | 94.1 | 1.1 | 5.6 | 93.2 | 1.2 | 5.1 | 93.7 | 48.6 | 51.4 |
| Education (highest level) | | χ^2^ (2) = 76.89*** | | | χ^2^ (2) = 15.49 | | | χ^2^ (2) = 24.30** | | | χ^2^ (2) = 26.94** | | | χ^2^ (2) = 35.49*** | | | χ^2^ (2) = 58.68*** | χ^2^ (2) = 102.18*** |
|  | 8th grade | 6.9 | 14.7 | 78.4 | 5.0 | 4.0 | 91.1 | 2.0 | 3.9 | 94.1 | 1.0 | 5.9 | 93.1 | 1.0 | 8.0 | 91.0 | 33.3 | 58.8 |
|  | High school | 3.3 | 10.2 | 86.5 | 2.3 | 8.5 | 89.2 | 0.5 | 3.9 | 95.7 | 0.7 | 4.4 | 94.9 | 0.7 | 4.1 | 95.2 | 48.5 | 59.5 |
|  | CDA | 4.4 | 13.7 | 81.9 | 1.8 | 7.7 | 90.5 | 1.0 | 3.3 | 95.7 | 1.1 | 3.4 | 95.5 | 0.9 | 4.0 | 95.1 | 45.1 | 60.2 |
|  | Associate degree | 4.0 | 10.3 | 85.7 | 2.8 | 8.9 | 88.4 | 1.0 | 4.4 | 94.5 | 1.2 | 5.2 | 93.5 | 1.6 | 5.2 | 93.3 | 51.4 | 53.9 |
|  | Bachelor’s degree | 2.6 | 6.9 | 90.5 | 3.3 | 8.3 | 88.5 | 1.1 | 3.8 | 95.0 | 1.7 | 4.8 | 93.5 | 1.6 | 4.2 | 94.3 | 55.0 | 50.9 |
|  | Graduate degree | 3.1 | 7.3 | 89.6 | 2.9 | 8.8 | 88.3 | 1.4 | 4.7 | 93.9 | 1.4 | 5.7 | 92.3 | 1.9 | 5.1 | 93.0 | 51.7 | 48.1 |
| Years in childcare | | χ^2^ (2) = 45.46*** | | | χ^2^ (2) = 16.50* | | | χ^2^ (2) = 6.44 | | | χ^2^ (2) = 4.48 | | | χ^2^ (2) = 5.87 | | | χ^2^ (2)  = 13.94** | χ^2^ (2) = 24.39*** |
|  | < 6 years | 2.9 | 8.5 | 88.6 | 2.9 | 8.7 | 88.5 | 0.8 | 3.9 | 95.3 | 1.1 | 4.5 | 94.4 | 1.2 | 4.2 | 94.7 | 51.8 | 56.8 |
|  | 6-10 years | 3.1 | 10.1 | 86.8 | 2.9 | 8.8 | 88.3 | 1.0 | 4.4 | 94.6 | 1.4 | 5.2 | 93.4 | 1.2 | 5.0 | 93.8 | 49.1 | 53.1 |
|  | 11-15 years | 4.0 | 12.7 | 83.3 | 2.0 | 9.3 | 88.7 | 1.1 | 5.1 | 93.9 | 1.0 | 5.4 | 93.7 | 1.0 | 5.3 | 93.8 | 46.8 | 49.8 |
|  | >15 years | 4.9 | 10.3 | 84.7 | 2.1 | 6.4 | 91.5 | 0.8 | 3.4 | 95.8 | 1.2 | 4.8 | 94.0 | 1.4 | 4.2 | 94.3 | 48.8 | 53.5 |
| Childcare setting | | χ^2^ (2) = 11.21 | | | χ^2^ (2) = 14.63 | | | χ^2^ (2) = 20.84* | | | χ^2^ (2) = 10.05 | | | χ^2^ (2) = 9.90 | | | χ^2^ (2)  = 6.17 | χ^2^ (2) = 39.63*** |
|  | Commercial | 3.2 | 9.1 | 87.7 | 3.2 | 8.9 | 87.9 | 1.0 | 4.2 | 94.9 | 1.2 | 4.2 | 94.5 | 1.2 | 4.6 | 94.2 | 50.1 | 57.8 |
|  | Non-Commercial | 3.5 | 9.8 | 86.7 | 2.5 | 8.0 | 89.5 | 0.6 | 3.7 | 95.7 | 1.0 | 4.8 | 94.2 | 1.0 | 4.3 | 94.7 | 51.0 | 54.5 |
|  | Religious | 2.6 | 8.7 | 88.8 | 1.6 | 9.1 | 89.2 | 1.1 | 3.0 | 95.9 | 1.3 | 4.1 | 94.6 | 1.2 | 3.6 | 95.2 | 52.0 | 54.0 |
|  | Head start | 3.4 | 10.1 | 86.5 | 2.8 | 7.2 | 90.0 | 0.7 | 3.9 | 95.4 | 1.1 | 4.4 | 94.5 | 1.2 | 4.0 | 94.8 | 50.2 | 57.0 |
|  | Home based | 3.8 | 10.6 | 85.6 | 2.6 | 8.2 | 89.1 | 0.7 | 5.3 | 94.0 | 0.9 | 5.4 | 93.7 | 0.7 | 5.3 | 94.0 | 46.6 | 61.9 |
|  | Other | 3.1 | 8.2 | 88.8 | 3.0 | 8.7 | 88.3 | 1.4 | 4.5 | 94.2 | 1.5 | 5.3 | 93.2 | 1.6 | 4.7 | 93.8 | 50.9 | 50.8 |
| Prior training | | χ^2^ (2) = 7.30* | | | χ^2^ (2) = 21.34*** | | | χ^2^ (2) = 28.69*** | | | χ^2^ (2) = 30.76*** | | | χ^2^ (2) = 34.66*** | | | χ^2^ (1)  = 0.76 | χ^2^ (1) = 37.07*** |
| Yes | | 3.6 | 9.5 | 86.9 | 3.0 | 8.9 | 88.0 | 1.1 | 4.5 | 94.4 | 1.4 | 5.3 | 93.4 | 1.4 | 5.0 | 93.6 | 50.9 | 53.4 |
| No | | 2.8 | 8.9 | 88.4 | 2.0 | 7.4 | 90.6 | 0.5 | 3.0 | 96.5 | 0.7 | 3.6 | 95.7 | 0.7 | 3.2 | 96.1 | 50.0 | 59.0 |

Note: CDA = Child Development Associate credential

*p-value<.05; **p-value<0.01; ***p-value<0.001

**Appendix Table 10**. Item level indicators of appropriateness by learner characteristic

| Endorsed program component as helpful | | | Resource documents | Explanation of legal requirements | Background stories | Available online at any time | Reporting procedures | |
| --- | --- | --- | --- | --- | --- | --- | --- | --- |
| Gender | | | χ^2^ (1)=2.24 | χ^2^ (1)=3.22 | χ^2^ (1)=4.81* | χ^2^ (1)=1.51 | χ^2^ (1)=15.38*** | |
|  | Female | | 35.5 | 34.5 | 23.4 | 25.2 | 34.3 | |
|  | Male | | 37.5 | 37.0 | 20.8 | 23.6 | 29.1 | |
| Race/ethnicity | | | χ^2^ (4)=3.62 | χ^2^ (4)=2.54 | χ^2^ (4)=64.8*** | χ^2^ (4)=10.1* | χ^2^ (4)=31.8*** | |
|  | | White | 35.3 | 34.5 | 21.1 | 25.4 | 32.4 | |
|  | | Asian | 36.9 | 34.7 | 25.9 | 30.3 | 40.2 | |
|  | | Hispanic | 36.6 | 36.7 | 26.2 | 22.8 | 39.8 | |
|  | | Black | 36.6 | 35.0 | 28.1 | 23.9 | 36.0 | |
|  | | Other | 39.7 | 37.7 | 27.0 | 21.7 | 35.7 | |
| Age | | | χ^2^ (2)=19.64*** | χ^2^ (2)=137.3*** | χ^2^ (2)=78.7*** | χ^2^ (2)=59.0*** | χ^2^ (2)=36.1*** | |
|  | | >45 years | 33.8 | 30.8 | 26.5 | 21.9 | 32.7 | |
|  | | 18-29 years | 37.0 | 34.5 | 19.3 | 27.7 | 31.7 | |
|  | | 30-44 years | 38.1 | 42.9 | 20.6 | 28.0 | 38.1 | |
| Education (highest level completed) | | | χ^2^ (5)=113.68*** | χ^2^ (5)=48.71*** | χ^2^ (5)=51.17*** | χ^2^ (5)=132.85*** | χ^2^ (5)=23.29*** | |
|  | | High school | 20.6 | 22.5 | 21.6 | 17.7 | 26.5 | |
|  | | 8th grade | 31.2 | 32.1 | 25.4 | 20.2 | 35.3 | |
|  | | CDA | 36.2 | 35.5 | 24.8 | 25.0 | 34.4 | |
|  | | Associate degree | 37.5 | 35.5 | 23.4 | 28.9 | 35.5 | |
|  | | Bachelor’s degree | 39.4 | 36.7 | 21.5 | 28.0 | 31.9 | |
|  | | Graduate degree | 43.2 | 40.4 | 16.8 | 32.0 | 30.2 | |
| Years as childcare professional | | | χ^2^ (3)=13.72** | χ^2^ (3)=55.87*** | χ^2^ (3)=14.80** | χ^2^ (3)=57.85*** | χ^2^ (3)=3.78 | |
|  | | < 6 years | 34.7 | 32.9 | 24.1 | 23.0 | 34.2 | |
|  | | 6-10 years | 37.2 | 35.9 | 22.4 | 26.9 | 32.4 | |
|  | | 11-15 years | 35.8 | 35.7 | 19.5 | 27.2 | 32.0 | |
|  | | >15 years | 39.0 | 42.1 | 21.3 | 31.0 | 34.4 | |
| Childcare setting | | | χ^2^ (5)=30.74*** | χ^2^ (5)=42.86*** | χ^2^ (5)=31.31*** | χ^2^ (5)=55.45*** | χ^2^ (5)=2.06 | |
|  | | Commercial | 33.0 | 32.0 | 25.8 | 22.0 | 33.5 | |
|  | | Non-commercial | 34.8 | 34.4 | 22.5 | 23.9 | 33.5 | |
|  | | Religious | 38.1 | 39.8 | 19.2 | 28.1 | 35.3 | |
|  | | Head start | 38.4 | 34.4 | 25.4 | 24.6 | 34.4 | |
|  | | Home based | 34.9 | 30.6 | 24.0 | 25.3 | 35.4 | |
|  | | Other | 39.4 | 38.5 | 21.4 | 30.2 | 32.9 | |
| Prior training | | | χ^2^ (1)=0.13 | χ^2^ (1)=4.03* | χ^2^ (1)=2.44 | χ^2^ (1)=56.29*** | χ^2^ (1)=39.10*** | |
|  | No | | 35.5 | 33.6 | 23.9 | 20.9 | | 37.5 |
|  | Yes | | 35.8 | 35.4 | 22.7 | 25.0 | | 31.9 |

Note: CDA = Child Development Associate credential; *p-value<.05; **p-value<.01; ***p-value<.00
